# Enhancing corn industry sustainability through deep learning hybrid models for price volatility forecasting

**DOI:** 10.1371/journal.pone.0323714

**Published:** 2025-06-09

**Authors:** Chengjin Yang, Yanzhong Zhai, Zehua Liu

**Affiliations:** School of Electronic Information Engineering, North China Institute of Science and Technology, Beijing, China; University of Queensland - Saint Lucia Campus: The University of Queensland, AUSTRALIA

## Abstract

The fluctuations in corn prices not only increase uncertainty in the market but also affect farmers’ planting decisions and income stability, while also impeding crucial investments in sustainable agricultural practices. Collectively, these factors jeopardize the long-term sustainability of the corn sector. In order to address the challenges posed by maize price volatility to the sustainability of the industry, this study proposes a multi-module wavelet transform-based fusion forecasting model: the TLDCF-TSD-BiTCEN-BiLSTM-FECAM (TLDCF-TSD-BBF) model, which is capable of accurately predicting short-term maize price volatility, thereby enhancing the sustainability of the industry. The model integrates a three-layer decomposition combined dual-filter time-series denoising method (TLDCF-TSD), a bidirectional time-convolutional enhancement network (BiTCEN), a bidirectional long- and short-term memory network (BiLSTM), and a frequency-enhanced channel attention mechanism (FECAM) to improve prediction accuracy and robustness. First, TLDCF-TSD is used to decompose the corn price time series into multiple scales, effectively separating the frequency components, extracting the signal details and trend information, and reducing the data complexity and non-stationarity. Secondly, BiTCEN designed in this paper effectively captures the short-term dependencies in the corn price data through the unique bidirectional structure and the special hybrid convolutional structure, and then accurately extracts the local features of the data, while BiLSTM mines the long-term trends and complex dependencies in the data by exploiting its bidirectional processing and long-term memory capabilities. Finally, FECAM enhances the focus on key temporal features in the frequency domain by grouping the input features along the channel dimensions and applying discrete cosine transform to generate attention vectors, improving the prediction accuracy and robustness of the model. The dataset utilized in this study was sourced from the BREC Agricultural Big Data platform, ensuring the reliability and accuracy of the corn price data for our analysis. This study utilizes price data from China’s five major corn-producing regions as a case study to demonstrate the efficacy of the proposed model in corn price forecasting. Through extensive experimentation, it has been established that the model significantly outperforms existing baseline models across various evaluation metrics. To be more specific, when dealing with different datasets, its MAE values are 0.0093, 0.0137, 0.0081, 0.0055, and 0.0101 respectively; the MSE values are 0.0002, 0.0002, 0.0001, 0.0001, and 0.0002 respectively; the MAPE values are 1.3630, 1.7456, 1.1905, 0.8456, and 1.7567 respectively; and the R2 values are 0.9891, 0.9888, 0.9943, 0.9955, and 0.9933 respectively. These data fully demonstrate the excellent performance of this model.

## Introduction

Corn, a key global crop, serves as a staple in many diets [1], and is rich in carbohydrates, proteins, vitamins, and minerals [2]. It plays a crucial role in livestock feed and is essential to industries such as bioenergy and chemical production [3], particularly in the production of ethanol, which significantly impacts economic and market stability [4]. As the global corn market continues to evolve, accurate price forecasting becomes increasingly vital for farmers, policymakers, and traders [5]. Price volatility not only affects economic profits and market stability [6] but also poses challenges to the sustainability of the corn industry. Developing an efficient and accurate prediction model is essential for understanding market trends, guiding agricultural decisions, and fostering the long-term sustainability of the corn sector. Predicting corn prices presents a multitude of challenges, such as market complexity, data incompleteness, seasonal fluctuations, and difficulties in ensuring model robustness and clarity [7]. The existence of these challenges confers a certain degree of unpredictability to corn prices. Therefore, for the agricultural sector, the development of models that can accurately forecast corn prices is of critical importance. Such models are not only instrumental in informing agricultural policy but also pivotal for the sustainable development of the corn sector [8].

Currently, the prediction of agricultural product prices primarily employs three predominant methodologies: traditional econometric statistical techniques, deep learning, and ensemble models. These approaches are designed to enhance the accuracy and robustness of forecasts, thereby equipping stakeholders to navigate market fluctuations and inform decision-making processes effectively [9].

Common traditional econometric statistical techniques include the Autoregressive Integrated Moving Average (ARIMA) [10], Generalized Autoregressive Conditional Heteroskedasticity (GARCH) [11], Error Correction Model (ECM) [12], and Vector Autoregression (VAR) [13]. Some economists used the ARIMA model to predict short-term cabbage prices with notable success [14]. Some researchers applied ARIMA for short-term price forecasting of Yunnan Pu-erh tea [15]. While traditional econometric methods can provide accurate predictions under the assumptions of linearity and stationarity, they face significant limitations in predicting the nonlinear and non-stationary nature of actual agricultural price series.

With advancements in computer technology, deep learning models [16] have become increasingly prevalent in agricultural price forecasting. These models can handle large datasets and learn complex patterns of price fluctuations. Recurrent Neural Networks (RNN) and Long Short-Term Memory networks (LSTM) [17,18] are particularly notable for their ability to capture time series features. LSTM models, by introducing gating mechanisms, effectively address the gradient vanishing problem in RNNs, improving prediction accuracy. Areef M and colleagues modeled and predicted potato prices using both GARCH and Artificial Neural Network (ANN) models, finding that the ANN model’s predictions were closer to actual prices [19]. Gu Yeong Hyeon proposed a Du-al-Input Attention LSTM (DIA-LSTM) model, which enhanced agricultural price forecasting performance [20]. Some young scholars compared LSTM and RNN models for soybean price prediction, finding that LSTM provided superior results [21].

The complexity of agricultural product price fluctuations means that a single model may be insufficient to accurately predict all types of market changes. To overcome the limitations of individual models, researchers have explored hybrid models or ensemble methods [22–24] that combine the strengths of multiple models to capture both linear and nonlinear features in price series. These hybrid models leverage the advantages of various predictive techniques, improving accuracy and robustness.

For example, Singla *et al*. proposed a solar irradiance prediction model combining iterative filtering with BiLSTM, demonstrating the value of combining data preprocessing with advanced deep learning networks in energy-related prediction, and providing a new idea for the study of agricultural price prediction [25]. A method combining Singular Spectrum Decomposition and LSTM-ARIMA for predicting hog prices was proposed in a published paper, achieving excellent performance [26]. The PCA-LSTM model, introduced by a researcher, excels at predicting Apple futures prices, offering superior fit and significant advantages in price prediction tasks [27]. The comprehensive prediction model (CEEMDAN-PCA-CNN-LSTM) for accurately forecasting the weekly average wholesale prices of pork in Henan Province was developed, integrating sequence decomposition, principal component analysis, and convolutional neural networks [28]. Despite these advancements, the application of advanced signal processing techniques and deep learning models [29] to agricultural price prediction remains relatively rare.

In recent years, a multitude of experts and scholars have delved into the realm of corn price prediction. A study utilized MATLAB to analyze the trajectory of Chinese corn prices from 2005 to 2016, employing both univariate nonlinear and multivariate linear regression models for forecasting [30]. Another investigation explored the application of neural network modeling to daily corn cash prices in U.S. markets, discovering that simple neural networks achieved high precision for one-day ahead predictions [31]. Despite these strides, the task of accurately predicting corn prices persists as a formidable challenge, largely due to the intrinsic complexity and volatility inherent in agricultural markets.

The innovations and features of this study are as follows:

1. The proposed TLDCF-TSD methodology effectively addresses the issue of complex frequency components and noise interference in corn price series. Specifically, the three-layer decomposition structure overcomes the inadequacy of the traditional decomposition in frequency separation, accurately distinguishing different frequency components, and dealing with the high-frequency part in depth to cope with the noise [32]. The dual-filter technique effectively removes the high-frequency noise and improves the stability of the data. The wavelet threshold denoising filters out noise in an accurate manner, based on the characteristics of the signal and the noise in the wavelet domain. Exponentially weighted moving average filtering then serves to smooth out the high-frequency component, thus reducing the complexity of the corn price series. This provides a solid data basis for accurate prediction of corn price. This results in a reduction in the complexity of the corn price series, thereby providing a robust data foundation for the accurate prediction of corn prices.
2. A novel hybrid model, designated BiTCEN-BiLSTM-FECAM, is put forth as a means of addressing the shortcomings of traditional models with respect to their inadequate feature extraction and information processing capabilities in the domain of corn price prediction. The BiTCEN is employed to extract the implicit relationships in the corn price series, and the extracted information is then input into the BiLSTM [33] for prediction. Finally, the FECAM is used to enhance the accuracy of the model by transforming the input features into the frequency domain and generating the attention vectors, which serve to further enhance the model’s attention to key temporal features and improve the model’s feature extraction ability in the frequency domain, thus improving the prediction accuracy. The hybrid model demonstrates excellent performance when applied to the corn price dataset of multiple provinces, thereby significantly enhancing the accuracy of corn price prediction and providing a more reliable basis for relevant decision-making.
3. The conventional time series analysis techniques frequently prove inadequate for fully elucidating the bidirectional temporal dependence inherent in corn price data. Additionally, there is a dearth of optimal utilisation of available information. The proposed BiTCEN method addresses the limitations of traditional methods [34], which are constrained to analysing data in a single direction. BiTCEN employs a bidirectional convolution structure, integrating information from both directions, and utilises a special activation function, rather than the traditional ReLu function, to enhance the network’s stability, adaptability and generalisation capabilities. Additionally, a hybrid structure combining ordinary convolution and dilated causal convolution is employed. These two components are complementary to one another, thereby enhancing the ability to extract complex features in the corn price data. This, in turn, facilitates more accurate price change pattern capture and provides more robust support for corn price prediction.

This study proposes a hybrid model based on wavelet transform, BiTCEN-BiLSTM-FECAM, for corn price prediction. The model incorporates several advanced modules. Extensive experiments demonstrate that our model significantly outperforms existing baseline models across multiple evaluation metrics in predicting corn prices across various provinces. This model not only provides a new perspective for corn price prediction but also serves as an effective tool for other agricultural price forecasts and time series analyses. Furthermore, the model’s insights into market dynamics can guide policy formulation, agricultural practices, and market interventions, ultimately contributing to food security and the sustainability of agricultural systems.

## Materials and methods

### Theoretical of multi-scale analysis

Multi-scale analysis is an essential technique for dissecting and understanding complex data structures, particularly in time series data. It operates by examining data across various scales, unearthing multi-level characteristics such as long-term trends, seasonal patterns, and short-term fluctuations. This approach is exceptionally beneficial for time-sensitive data like corn prices, where market dynamics and seasonal variations significantly influence price volatility. By applying multi-scale analysis, researchers can isolate and predict both the overarching trends and the subtle fluctuations that occur within shorter time frames.

The theoretical foundation of multi-scale analysis is anchored in mathematical tools such as wavelet transforms. These tools enable detailed scrutiny of data at different resolutions, revealing distinct patterns at various scales. The ability to analyze data at both coarse and fine scales simultaneously provides a comprehensive view of the data’s behavior over time. This dual-scale examination is crucial for complex systems, allowing for a more nuanced understanding of the interplay between long-term market trends and immediate price oscillations, as seen in agricultural commodities like corn.

The application of multi-scale analysis to corn price data confers numerous ad-vantages, primarily enhancing the accuracy and robustness of predictive models. By decomposing price data into features that represent different time scales, this technique offers deeper insights into the data’s dynamics. It empowers stakeholders with a clearer vision of market trends, informing strategic agricultural policies and market interventions. Ultimately, multi-scale analysis serves as a potent tool for predicting and navigating the complexities of time series data in various domains, including agriculture and finance.

### Basic module

#### Three-Layer Decomposition Combined Dual-Filter Time Series Denoising Method (TLDCF-TSD).

Time series data can usually be represented as y(t), where *t* represents time. Time series often contain multiple frequency components. The low-frequency part generally represents the long-term trend, while the high-frequency part contains more short-term fluctuations and noise. Through three-layer decomposition, the separation of different frequency components can be gradually refined. For discrete wavelet transform, let the original signal be f(t), and its discrete wavelet transform [35] is:

$$W_{f}(j,k)=\int_{-\infty }^{\infty }f(t)\;\phi_{j,k}(t)\;dt,$$ (1)

where $\phi_{j,k}(t)$ is the discrete wavelet function, usually expressed as $\phi_{j,k}(t)=j^{-1/2}\phi \left(\frac{t-k}{j}\right)$, where *j* and *k* are scale parameters and translation parameters, respectively. Discrete wavelet decomposition and reconstruction can be implemented via filter banks, with the formula as follows:

$$a[n]=\sum_{k}x[k]h[2n-k],$$ (2)

$$d[n]=\sum_{k}x[k]g[2n-k],$$ (3)

$$x[n]=\sum_{k}a[k]h^{\prime }[n-2k]+\sum_{k}d[k]g^{\prime }[n-2k].$$ (4)

where *x*[*n*] represents the original signal, *a*[*n*] and *d*[*n*] denote the low-frequency approximation coefficients and high-frequency detail coefficients, respectively. *h*[*n*] and *g*[*n*] serve as low-pass and high-pass filters, extracting the signal’s low and high-frequency components, while $h^{\prime }[n]$ and $g^{\prime }[n]$ are their corresponding inverse filters used for reconstruction.

Two-layer decomposition may not be sufficient to fully separate the different frequency components and accurately identify and process the noise in the high-frequency part. On the other hand, a higher number of layers could lead to excessive computational complexity without significantly improving the denoising effect. Three-layer decomposition strikes a balance between effectively separating the frequency components and maintaining computational efficiency. It allows for a more detailed analysis of the high-frequency components while not overly burdening the computational process. This makes it an optimal choice for this time series denoising method.

**Fig 1:**
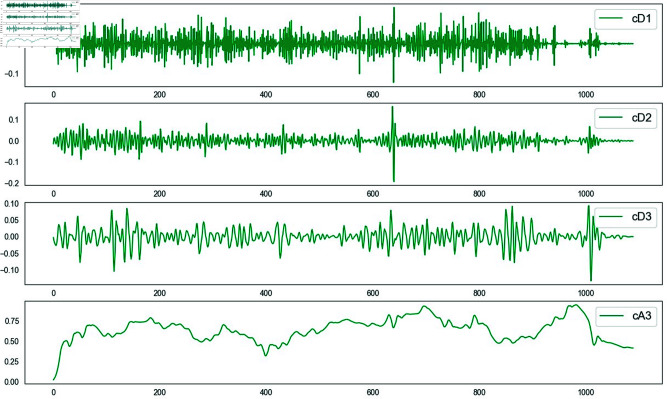
3-layer decomposition results of Daubechies wavelet function.

The first layer of decomposition initially distinguishes the low-frequency trend and high-frequency fluctuations, laying the foundation for more in-depth analysis later. The second layer of decomposition further refines the high-frequency part, enabling us to observe high-frequency features more carefully. The high-frequency detail component is further decomposed to obtain more subdivided high-frequency components. The third layer of decomposition conducts a more in-depth analysis of the high-frequency part, which is helpful for accurately identifying and processing noise.Compared with other numbers of layers, three-layer decomposition can achieve a good balance between denoising effect and computational efficiency while ensuring the full separation of different frequency components. This layering method conforms to the multi-resolution analysis theory in signal processing and can effectively decompose complex time series signals into relatively simple sub-signals, facilitating subsequent targeted processing [36]. The three-layer decomposition effect diagram is shown in Fig 1.

**Fig 2:**
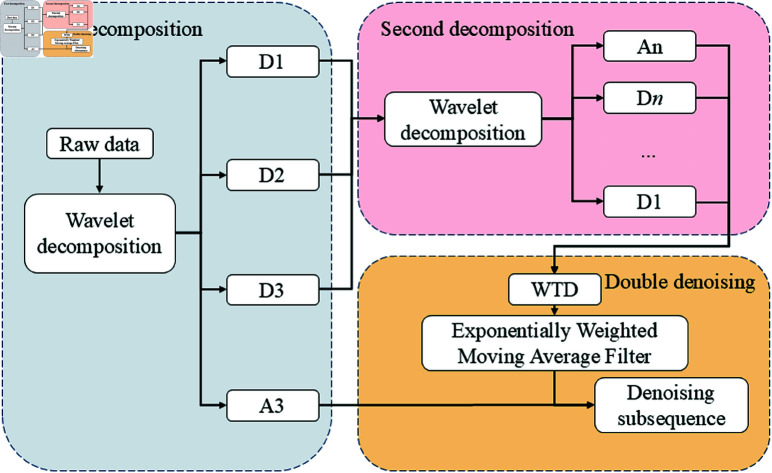
Flowchart of TLDCF-TSD.

The flow chart of three-layer decomposition combined with dual-filter time series denoising is shown in Fig 2. The high-frequency coefficient reconstruction concentrates the high-frequency components scattered by the wavelet decomposition into a single high-frequency component. Assume that the reconstructed high-frequency component is *HF*(*t*). Wavelet transform has good time-frequency localization characteristics. In the wavelet domain, signals and noise exhibit different characteristics. For wavelet transform, let the wavelet transform of the original signal *f*(*t*) be *Wf*(*a*,*b*), where *a* is the scale parameter and *b* is the translation parameter. Noise usually has smaller coefficients in the wavelet domain and is distributed more randomly, while the wavelet coefficients of the signal are relatively larger and have a certain structure. By setting an appropriate threshold for wavelet threshold denoising, the noise in the high-frequency component can be effectively removed while useful high-frequency information is retained. Let the threshold be T, and the wavelet coefficient after threshold processing be ${\tilde{W}}_{\text{HF}}(a,b)$. Then we have: when $|W_{\text{HF}}(a,b)|>T$, ${\tilde{W}}_{\text{HF}}(a,b)=W_{\text{HF}}(a,b)$; when $|W_{\text{HF}}(a,b)|\leq T$, ${\tilde{W}}_{\text{HF}}(a,b)=0$. This method is based on the theoretical basis of wavelet transform and can effectively remove the noise in the high-frequency part of the time series without excessive influence on the low-frequency trend component.

**Fig 3:**
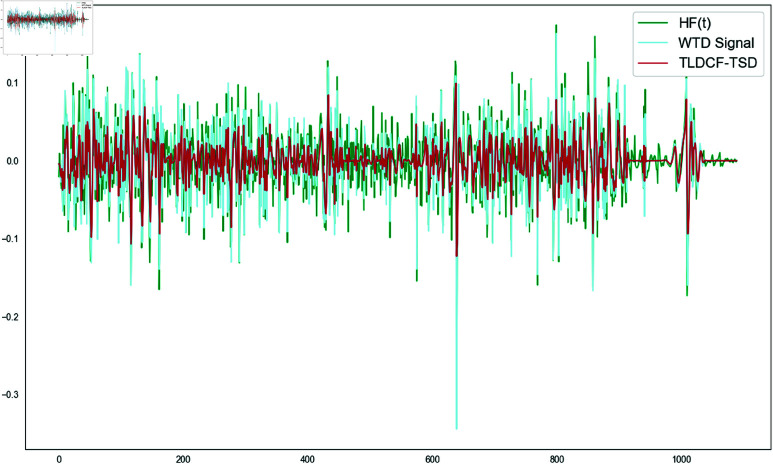
TLDCF-TSD denoising effect.

The exponentially weighted moving average filter gives higher weight to recent data and can quickly adapt to the dynamic changes of time series. After wavelet threshold denoising, applying exponentially weighted moving average filtering to the high-frequency component can further smooth the data, reduce the complexity of the high-frequency component, and make the data more stable and reliable. Let the high-frequency component after exponentially weighted moving average filtering be ${\text{EWMA}}_{\text{HF}}(t)$. Its calculation formula is

$${\text{EWMA}}_{\text{HF}}(t)=\alpha \cdot \text{HF}(t)+(1-\alpha )\cdot {\text{EWMA}}_{\text{HF}}(t-1)$$ (5)

where α is the smoothing parameter and takes values between 0 and 1. This filter design is in line with the characteristics of time series data and can effectively reduce noise and complexity while retaining the dynamic changes of time series. The denoising effect of TLDCF-TSD is shown in Fig 3.

Sample entropy is a characterization function used to measure the complexity of a sequence. Its value can intuitively reflect the complexity of the sequence. The smaller the sample entropy value, the higher the self-similarity of the sequence, which means that the sequence is less complex. The larger the value, the more complex the sequence. Sample entropy is represented by sample En(m, r, N), where N is the length of the data sequence, m is the dimension, and r is the similarity tolerance. Its formula is defined as follows:

$$\text{SampEn}(m,r)=\lim_{N\to \infty }\left(-\ln\left[\frac{B^{\left(m+1\right)}(r)}{B^{m}(r)}\right]\right)$$ (6)

When *N* is finite, the above definition formula of sample entropy can be expressed as:

$$\text{SampEn}(m,r,N)=-\ln\left[\frac{B^{\left(m+1\right)}(r)}{B^{m}(r)}\right]$$ (7)

In the above formula, *B*_*m* + 1_(*r*) and *B*_*m*_(*r*) are the probabilities of two time series matching m+1 or *m* points at the threshold *r*. Compared with approximate entropy, sample entropy has better consistency and is not sensitive to the loss of data sequences. It is more suitable for evaluating the complexity of sequences.

**Fig 4:**
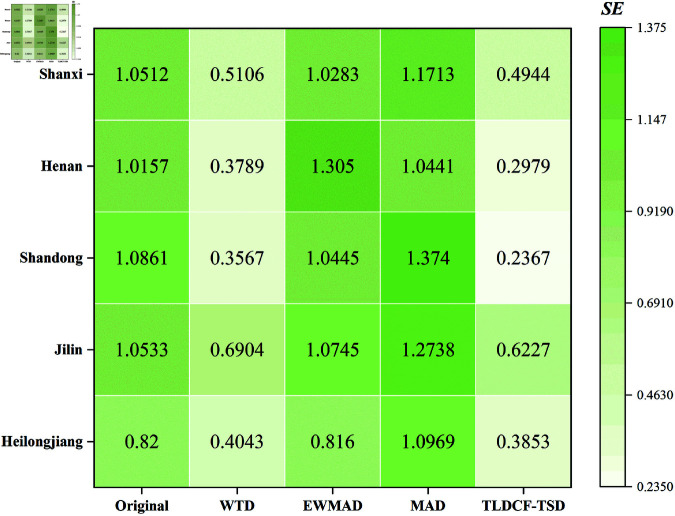
Sample entropy heatmap display of data complexity.

It can be clearly seen from Fig 4 that compared with several other denoising methods (wavelet threshold denoising, exponentially weighted moving average denoising, and moving average denoising), TLDCF-TSD can significantly reduce the non-stationarity of the sequence.

#### Bidirectional Temporal Convolutional Enhanced Network (BiTCEN).

A one-dimensional convolutional neural network (1D CNN) is a specialized type of convolutional neural network designed to extract local features from time series data by utilizing one-dimensional convolutional kernels along the time axis. Initially, the input data is processed through convolutional layers, where the kernels slide along the time axis and perform dot product operations with the input data, creating feature maps. These feature maps are then passed through non-linear activation functions, enhancing the model’s expressive capabilities. Stacking multiple convolutional layers enables the model to capture deeper temporal dependencies. Additionally, 1D CNNs may include pooling layers that reduce the dimensions of the feature maps by taking the maximum or average value within a local region, thereby decreasing computational complexity and mitigating overfitting risks. After multiple convolutional and pooling layers, the feature maps are integrated into a one-dimensional vector through a fully connected layer. Finally, the output layer performs predictions based on the task, such as classification or regression. This approach allows 1D CNNs to efficiently process one-dimensional time series data and extract complex temporal features. However, 1D CNNs may be less effective in capturing long-range dependencies.

Temporal Convolutional Network (TCN) [37] is a deep learning model composed of a series of causal convolutional layers and dilated convolutional layers. When applied to time-series tasks, TCN effectively maintains temporal order while leveraging parallel computation capabilities. Compared to 1D CNNs, TCN demonstrates improved stability and training efficiency, making it particularly suitable for handling long sequence data. However, TCN only employs forward convolutions, extracting features solely from the forward input sequence of corn prices, thereby overlooking implicit information in the backward sequence.

To address this limitation, we propose a new time series processing algorithm called Bidirectional Temporal Convolutional Enhanced Network (BiTCEN). The structural design of BiTCEN is unique and significantly different from traditional algorithms. Traditional bidirectional temporal convolutional networks have limitations when processing time series data. BiTCEN not only retains the bidirectional structure but also conducts in-depth optimization. By processing the time series through forward and backward temporal convolutional layers respectively and then cleverly fusing the outputs of the two directions, it can more comprehensively capture the long-term dependencies and dynamic changes in the time series. In terms of the activation function, LeakyReLu is used instead of the traditional ReLu. The LeakyReLu activation function allows a small non-zero slope when the input is negative, effectively alleviating the neuron “death” problem that may be caused by the traditional ReLu activation function, making the network more stable during training, better adapting to complex data distributions, and greatly improving the generalization ability of the model. At the same time, a hybrid structure combining ordinary convolution and dilated causal convolution is adopted. An ordinary convolution layer is added before the dilated causal convolution. Ordinary convolution can capture the local spatial correlation of the time series, and dilated causal convolution can extract features on different time scales, providing a powerful tool for feature extraction.

BiTCEN can extract richer and more accurate features from time series data. This performance surpasses many existing time series analysis algorithms. Through innovative designs of the bidirectional structure, activation function, and convolution combination, it can better adapt to the complexity and variability of time series data and provide more valuable information for subsequent analysis and prediction tasks. For example, in time series analysis in different fields, BiTCEN can more accurately capture key features and provide strong support for decision-making.

**Fig 5:**
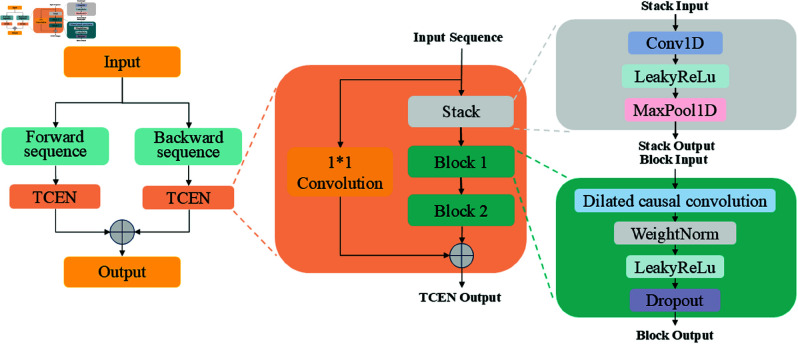
BiTCEN network structure.

BiTCEN has high flexibility and scalability. It can be adjusted and optimized according to different time series data types and analysis tasks. This characteristic enables BiTCEN to adapt to different application scenarios and provides more possibilities for research and application in the field of time series analysis. Whether it is price prediction in the financial field, forecasting in the meteorological field, or equipment monitoring in the industrial field, BiTCEN can play an important role and provide new ideas and methods for solving practical problems. The structure of BiTCEN is illustrated in Fig 5.

#### Bidirectional Long Short-Term Memory Network (BiLSTM).

RNN is a neural network structure designed for sequential data processing. Unlike traditional feedforward neural networks, RNNs feature recurrent connections that al-low them to retain previous input information and use it in current outputs. This capability makes RNNs particularly suitable for handling time-series data or data with sequential relationships. In RNNs, the recurrent connections in the hidden layers combine the current input with the previous hidden state at each time step *t*, enabling the network to capture temporal dependencies in the data. However, standard RNNs often encounter issues such as gradient vanishing or exploding gradients when processing long sequences, limiting their ability to capture long-term dependencies effectively.

LSTM addresses these limitations as an improved version of RNNs by introducing memory cells and gate mechanisms to control information storage and flow. LSTM consists of a series of memory cells and three types of gates: the input gate, the forget gate, and the output gate. The core idea is to facilitate interactions among these gates to manage long-term dependencies efficiently. Specifically, the input gate regulates new information entering the recurrent network, the forget gate controls how much historical information to discard, and the output gate selects the most relevant information for the current time step prediction and outputs it.

**Fig 6:**
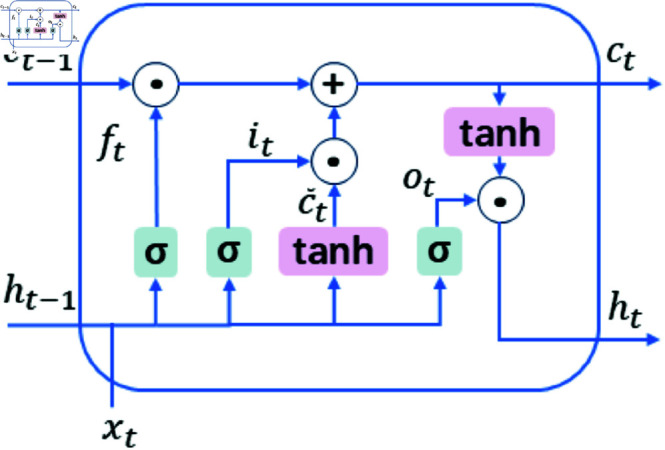
LSTM architecture.

LSTM computes three values between 0 and 1 using fully connected layers with sigmoid activation functions, which determine the input gate, forget gate, and output gate values based on the current input and the previous hidden state. The temporary memory cell values are computed using the tanh activation function and depend on how much content from the temporary memory cell the input gate adopts, while the forget gate retains content from the previous time step memory cell. The hidden state controls the output of the memory cell through the output gate. The computations are as follows:

$$f_{t}=\sigma (W_{hf}h_{t-1}+W_{xf}x_{t}+b_{f})$$ (8)

$$i_{t}=\sigma (W_{hi}h_{t-1}+W_{xi}x_{t}+b_{i})$$ (9)

$$o_{t}=\sigma (W_{ho}h_{t-1}+W_{xo}x_{t}+b_{o})$$ (10)

$${\tilde{c}}_{t}=\tanh(W_{hc_{t}}h_{t-1}+W_{xc_{t}}x_{t}+b_{c_{t}})$$ (11)

$$c_{t}=f_{t}⊙c_{t-1}+i_{t}⊙{\tilde{c}}_{t}$$ (12)

$$h_{t}=o_{t}⊙\tanh(c_{t})$$ (13)

where *f*_*t*_ represents the forget gate, *i*_*t*_ is the input gate, *o*_*t*_ is the output gate, ${\tilde{c}}_{t}$ is the temporary memory cell, *c*_*t*_ is the memory cell, *h*_*t*_ is the hidden state, *W*_*hf*_, *W*_*xf*_, *W*_*hi*_, *W*_*xi*_, *W*_*ho*_, *W*_*xo*_, $W_{hc_{t}}$, $W_{xc_{t}}$ denote the weight parameters, and *b*_*f*_, *b*_*i*_, *b*_*o*_, $b_{c_{t}}$ are the bias parameters. The architecture of LSTM is illustrated in Fig 6.

**Fig 7:**
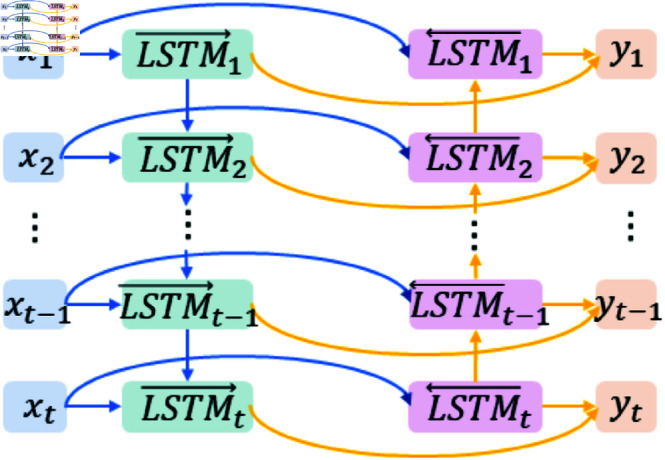
BiLSTM architecture.

BiLSTM is an advanced form of recurrent neural network designed to handle and predict time series data. It combines two LSTM networks: one processes the sequence from start to end (forward LSTM), and the other processes it from end to start (backward LSTM). This bidirectional structure allows BiLSTM to leverage both past and future context at each time step, resulting in a more comprehensive understanding of the data. The architecture of BiLSTM is illustrated in Fig 7.

In addition, in the BiLSTM model, the input gate is calculated by the sigmoid activation function to give the input gate value *i*_*t*_, which determines how much new input information *x* can enter the memory unit for updating at the current time *t*. If the input threshold is close to 1, more new information is allowed to enter; if it approaches 0, new information is prevented from entering, thus preventing irrelevant or incorrect information from interfering with model learning and reducing the introduction of errors. The forget gate also uses the sigmoid activation function to calculate the forget gate value *f*_*t*_, which is used to determine how much information *c*_*t*−1_ was retained in memory at the previous instant. If the forget gate value is close to 1, more historical information will be retained; if it is close to 0, more historical information will be forgotten. This helps the model to dynamically adjust its reliance on historical information when processing long sequence corn price data, preventing outdated or erroneous historical information from continuing to influence subsequent forecasts, and correcting for errors that may be caused by the accumulation of historical information. The output gate calculates the output gate value *o*_*t*_ through the sigmoid activation function and determines the output *h*_*t*_ of the model at the current time by combining the activation function tanh with the processed storage unit information $\tanh(c_{t})$. The output gate can select the most relevant and reliable information as the output based on the current input and memory status, thereby avoiding the output of irrelevant or erroneous information and further reducing error propagation.

Introducing the BiLSTM module into the model architecture and integrating a backpropagation algorithm and gradient pruning strategy to effectively suppress the error propagation and amplification effects caused by gradient-related problems. With this mechanism, it is possible to ensure that the model continuously approaches the optimal solution during the training process, thereby achieving accurate correction of prediction errors, significantly improving the accuracy and reliability of model predictions, and enhancing the generalisation ability and robustness of the model in complex data environments.

#### Frequency Enhanced Channel Attention Mechanism (FECAM).

**Fig 8:**
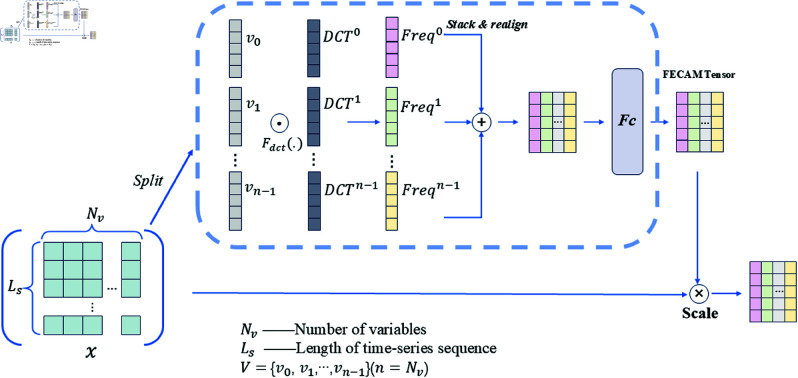
Structure of frequency enhanced channel attention mechanism.

FECAM [38] is a method that enhances attention by splitting input features along the channel dimension into multiple subgroups and applying Discrete Cosine Transform (DCT) [39] to each subgroup, generating an attention vector. Although FECAM is not the direct product of the transformer model, it does adopt the core idea of the attention mechanism and extends it to meet the specific needs of time series analysis. By enhancing the expression of features in the frequency domain, FECAM provides a new perspective and method for time series prediction. The structure of FECAM is illustrated in Fig 8. Specifically, the input time series data *x* is divided into *n* subgroups, each subgroup $V_{i}$, i∈{0,1,⋯,n−1}, $n=N_{v}$, undergoes DCT, calculated as follows:

$$B_{l}^{j}=\cos\left(\frac{\pi l}{L_{s}}\left(i+\frac{1}{2}\right)\right)$$ (14)

$${\text{Freq}}^{i}={\text{DCT}}^{j}(V_{i})=\sum_{j=0}^{L_{s}-1}(V_{i,l})B_{l}^{j}$$ (15)

where $l\in \{0,1,\cdots ,L_{s}\;-\;1\}$, $B_{l}^{i}$ represents the DCT basis function, and ${\text{DCT}}^{j}$ denotes the *j*-th coefficient obtained from applying the DCT to $V_{i}$. The time-domain signal $V_{i}$ is transformed into its frequency-domain representation using DCT, where each ${\text{DCT}}^{j}$ corresponds to a frequency component in the frequency domain. The process of converting data from the time domain to the frequency domain and stacking it to form a frequency domain vector is as follows:

$$\text{Freq}=\text{DCT}(V)=\text{stack}([{\text{Freq}}^{0},{\text{Freq}}^{1},\cdots ,{\text{Freq}}^{n-1}])$$ (16)

The transformed frequency-domain data is then normalized. Using a fully connected neural network Fc, frequency-domain vector data is generated to adjust the previously defined attention weights. Finally, the input data is multiplied by the adjusted attention weights to produce the final output. The calculation process is as follows:

$$F_{\text{c-att}}=\sigma (W_{2}\delta (W_{1}\text{DCT}(V)))$$ (17)

$$\text{Output}=V\times F_{\text{c-att}}$$ (18)

where *W*_1_ and *W*_2_ are the weight matrices of the fully connected layers, and $F_{\text{c-att}}$ represents the generated attention weights, reflecting the importance of different channels at various frequencies. This process allows each channel’s features to interact with all frequency components, effectively capturing critical time series features and improving the model’s feature extraction and generalization capabilities.

Compared to traditional attention mechanisms, such as channel attention mechanisms, FECAM’s main advantage lies in its ability to more effectively capture and enhance important frequency components in the input data. Traditional attention mechanisms typically focus on spatial or temporal features, while FECAM, by incorporating DCT, converts input data from the time domain to the frequency domain, thus enhancing attention to frequency information. Since some crucial features in time series data are difficult to extract in the time domain and are often present in specific frequency patterns, FECAM can more accurately capture these key features, improving the model’s performance on complex tasks.

### TLDCF-TSD -BiTCEN-BiLSTM-FECAM (TLDCF-TSD -BBF) prediction model

**Fig 9:**
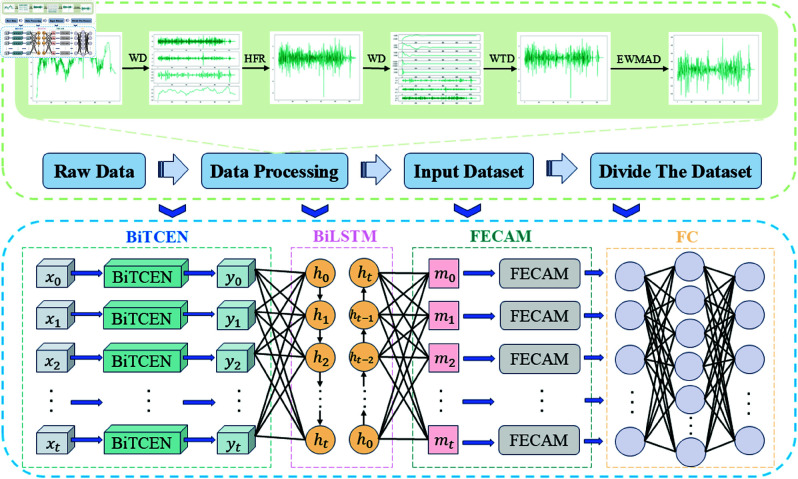
Structure of TLDCF-TSD -BiTCEN-BiLSTM-FECAM prediction model.

The hybrid TLDCF-TSD-BBF model for corn price prediction comprises four key modules: TLDCF-TSD, BiTCEN, BiLSTM, and FECAM. As shown in Fig 9, this process begins with TLDCF-TSD. It performs multi-scale decomposition and reconstruction on the input time series. By means of double filtering, it reduces the complexity and non-stationarity of the time series, thereby improving the prediction ability and accuracy of future price changes. Next, BiTCEN uses dilated convolutions to expand the receptive field, capturing short-term dependencies and leveraging both forward and backward information for enhanced feature extraction. Following this, BiLSTM processes the sequence bidirectionally to capture long-term dependencies. By positioning BiTCEN ahead of BiLSTM, the configuration capitalizes on BiTCEN’s proficiency in extracting local features and short-term dependencies, while leveraging BiLSTM’s aptitude for managing long-term dependencies and contextual information. Serving as the preliminary module, BiTCEN employs its convolutional architecture to swiftly process data in parallel, detecting high-frequency fluctuations within time series and concurrently mitigating computational load and the vanishing gradient issue. Subsequently, BiLSTM, as the subsequent module, utilizes its gating mechanisms to thoroughly analyze the features extracted by BiTCEN, integrating long-term dependencies and enhancing the model’s comprehension and predictive capacity for time series. Finally, FECAM applies frequency domain processing to the extracted features, enhancing attention to critical characteristics. The model maps these key features to a one-dimensional space through a fully connected layer to generate the final output. By integrating multi-scale feature extraction, short-term and long-term information processing, and frequency domain enhancement, this model significantly improves the accuracy of corn price predictions.

Overall, the models proposed in this study make innovative contributions in various aspects. The TLDCF-TSD method effectively overcomes the shortcomings of traditional methods in dealing with frequency components and noise in maize price data through its unique three-layer decomposition structure and double filtering technique. It separates frequency components, removes noise and smoothes the data, providing a more stable and higher quality data base for the model, which is not achievable with traditional models. The BiTCEN - BiLSTM - FECAM hybrid model innovatively combines the benefits of multiple network structures. BiTCEN, with its bidirectional convolutional structure, special activation function and convolutional combinations, is able to extract short-term dependencies and local features. It comprehensively captures dependencies and extracts complex features, reducing error accumulation. BiLSTM, by managing information through gating mechanisms and combining with features extracted from BiTCEN, is good at mining long-term trends and complex dependencies. It controls error propagation and further improves the model’s understanding of the data. Through the frequency domain attention mechanism, FECAM improves the expression of key features, optimises model decision making and reduces errors caused by feature extraction problems.

In addition, through the design of the bidirectional convolutional structure and special activation function, BiTCEN breaks the limitations of traditional time series analysis techniques in capturing bidirectional time dependence and exploiting information. It better adapts to the complexity of maize price data, providing strong support for accurate forecasting. Together, these strategies significantly improve the model’s feature extraction capability and forecasting accuracy on corn price data, outperforming traditional single models or simple combination models.

## Experiments setting

### Data preparation

The data selected for this study comes from the BREC Agricultural Big Data platform, which covers corn price data from 1 January 2021 to 2 January 2023 in the five major corn producing regions of Heilongjiang, Shanxi, Henan, Jilin, and Shandong. These regions play an important role in national corn production, and their price data is representative of the overall trend and characteristics of China’s corn market, so they were selected as the research object. Table 2 presents the statistical information on corn prices from 2021 to 2023 for the provinces of Heilongjiang, Shanxi, Henan, Jilin, and Shandong. Each province has its own highest, lowest, and average values, reflecting differences in price levels and fluctuation ranges. From a statistical perspective, the skewness is negative, indicating that the data is skewed to the left. The kurtosis indicates that the data distribution has varying degrees of peak or flatness. The J-B statistic is relatively large, indicating that the data significantly deviates from a normal distribution. The huge Q(20) value means that the autocorrelation of the corn price time series is extremely strong when lagged by 20 orders, which provides important basis for subsequent model construction and analysis.

In terms of data processing, we select the training and test data for this study based on a variety of theoretical and practical considerations. On the one hand, based on the classical theory of data partitioning in the field of time series analysis, as well as the existing literature and research results in related fields, the selected data is partitioned into training and test sets in the ratio of 8:2, where 80% is used to train the model and 20% is used to test its performance. This ratio has been proven in many previous studies to ensure that the model can fully learn the features of the data, while the test set is used to effectively evaluate the generalisation ability of the model, thus ensuring the reliability of the model performance evaluation. On the other hand, from the theory of data timeliness and appropriateness, the data of this period is selected because of its remarkable timeliness, which can comprehensively cover the recent market changes, and its time-series characteristics are highly compatible with the multi-scale analysis and time-series forecasting model of this study, which can help to delve deeply into the dynamic change law of maize price, thus ensuring the timeliness and validity of the study.

To ensure data quality and consistency, we preprocessed the raw data by cleaning it, removing outliers and missing values, and normalizing the price data using Min-Max normalization to scale all features to the [0, 1] range. The normalization formula is as follows:

$$X^{*}=\frac{X-X_{\min}}{X_{\max}-X_{\min}}$$ (19)

where ${\text{X}}^{*}$ is the normalized value of the data, *X* is the original data value, $X_{\min}$ is the minimum value in the data, and $X_{\max}$ is the maximum value in the data.

Min-Max normalisation is based on the theory of data normalisation, which eliminates the difference in magnitude of values between different features due to the difference in scale by mapping the original data to the interval [0,1] so that the data is processed on the same scale. This helps to improve the stability and speed of model training, as data at different scales can lead to unstable gradient updates during model training, and normalisation allows the gradient descent algorithm to converge to the optimal solution more quickly. At the same time, normalisation also improves the generalisability of the model and avoids over-reliance of the model on specific scales of data, making it more adaptable to different datasets.

**Table 1:** Corn Price Data for Heilongjiang, Shanxi, Henan, Jilin, and Shandong Provinces from 2021 to 2023.

Province	Maximum	Minimum	Average	Skewness	Kurtosis	J-B	Q(20)
Heilongjiang	2958.00	2250.00	2655.43	-0.40	0.14	30.41^***^	15155.15^***^
Jilin	2923.00	2379.00	2730.97	-0.66	-0.06	79.99^***^	12992.65^***^
Shandong	2992.00	2444.00	2816.23	-0.70	2.32	330.86^***^	12183.60^***^
Henan	3082.00	2454.00	2865.68	-0.98	1.93	343.71^***^	11684.05^***^
Shanxi	2993.00	2492.00	2810.95	-0.41	0.87	64.73^***^	11657.12^***^

### Experimental environment and parameter setting

In this paper, the deep learning framework PyTorch is used to build the environment required for the simulation experiments. The specific parameters of the environment are CPU, Intel(R) Core(TM) i5-8250U CPU @ 1.60GHz 1.80GHz; RAM, 20.0 GB; Operating system, Windows 11, 64-bit; PyTorch version 2.2.1; Python version 3.11.8. Simultaneously, the parameters were optimised using the Adam optimiser, with the mean square error (MSE) selected as the loss function. Furthermore, in order to ascertain the optimal model parameter configurations, a reasonable search space is initially established for each hyperparameter to encompass the most favourable configuration. Following a series of iterative processes and rigorous verification, the combination of hyperparameters that can lead to optimal model performance on data from diverse geographical regions was ultimately identified. The specific hyperparameter settings are delineated in Table 2.

**Table 2:** Parameter setting.

Hyperparameters	Optimum
Learning rate	0.001
Epochs	800
Number of layers (BiLSTM)	5
Number of Hidden Nodes (BiLSTM)	70
Optimizer	Adam

### Evaluation metrics

In this study, we employed several evaluation metrics, including MAE, MSE, Mean Absolute Percentage Error (MAPE), and Coefficient of Determination (R-squared), to comprehensively assess the performance of the models and accurately quantify improvements across different models.

MAE measures the average absolute difference between predicted values and actual values. A lower MAE indicates more accurate predictions. The formula is as follows:

$$\text{MAE}=\frac{1}{n}\sum_{i=1}^{n}\left|y_{i}-{\hat{y}}_{i}\right|$$ (20)

where *y*_*i*_ represents the actual values, ${\hat{y}}_{i}$ denotes the predicted values, and *n* signifies the sample count. MAE reflects the average magnitude of errors.

MSE measures the average squared difference between predicted values and actual values. A smaller MSE indicates more accurate predictions, though it is more sensitive to outliers compared to MAE. The formula is as follows:

$$\text{MSE}=\frac{1}{n}\sum_{i=1}^{n}{\left(y_{i}-{\hat{y}}_{i}\right)}^{2}$$ (21)

MAPE serves as a robust indicator of the relative error between predicted and actual values. A lower MAPE signifies a model with a higher degree of precision in its predictions, thereby enhancing the reliability of the forecast outcomes. The formula for MAPE is presented as follows:

$$\text{MAPE}=\frac{1}{n}\sum_{i=1}^{n}\left|\frac{y_{i}-{\hat{y}}_{i}}{y_{i}}\right|\times 100\%$$ (22)

The R-squared metric quantifies the proportion of the variance in the dependent variable that is predictable from the independent variables. An R-squared value that approaches unity indicates an excellent fit of the model to the data, suggesting a strong explanatory capacity. The R-squared is calculated using the formula:

$$R^{2}=1-\frac{\sum_{i=1}^{n}{\left(y_{i}-{\hat{y}}_{i}\right)}^{2}}{\sum_{i=1}^{n}{\left(y_{i}-{\bar{y}}_{i}\right)}^{2}}$$ (23)

where ${\bar{y}}_{i}$ is the mean of the actual values.

To ascertain whether there exists a statistically significant disparity in the predictive accuracy between two models under consideration, the DM (Diebold - Mariano) test is commonly employed. The DM test functions by initially constructing a sequence of differences in prediction errors. Given two models, say Model A and Model B, with prediction error sequences *e*_*A*,*t*_ and *e*_*B*,*t*_ respectively for t=1,2,…,T (where *T* represents the number of samples), the error difference sequence $d_{t}=e_{A,t}-e_{B,t}$ is formed.

The DM statistic is computed through a series of steps. First, the sample mean of the error difference sequence $\bar{d}=\frac{1}{T}\sum_{t=1}^{T}d_{t}$ is calculated, which provides an indication of the average difference in prediction errors. Subsequently, the variance of the error difference sequence ${\hat{\sigma }}_{d}^{2}$ is estimated. This is often achieved by leveraging the sample autocovariance function. Let $\hat{\gamma }(k)$ denote the sample autocovariance function of *d*_*t*_ at lag *k*, and the variance estimate is given by

$${\hat{\sigma }}_{d}^{2}=\hat{\gamma }(0)+2\sum_{k=1}^{q}\left(1-\frac{k}{q+1}\right)\hat{\gamma }(k),$$ (24)

where *q* is an appropriately selected lag order, which can be determined using information criteria such as AIC or BIC. Finally, the DM statistic is computed as

$$\text{DM}=\frac{\bar{d}}{\sqrt{\frac{{\hat{\sigma }}_{d}^{2}}{T}}}.$$ (25)

## Experiments and results

### Ablation experiment analysis

This study investigates the performance of various model combinations in a corn price forecasting context, focusing on the contribution of individual modules to the overall predictive accuracy. Utilizing data from key corn-producing regions in China, we demonstrate the superior predictive capabilities of the TLDCF-TSD-BBF model.

In our ablation experiments, we compared the performance of different model configurations to evaluate the enhancement effect of each module on the overall fore-casting performance. The experimental dataset encompassed corn price data from five major corn-producing provinces in China: Heilongjiang, Jilin, Shandong, Henan, and Shanxi.

**Fig 10:**
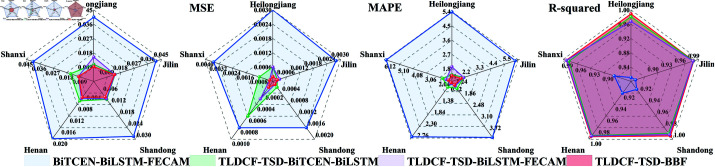
Comparison of accuracy indicators between ablation model and our proposed model.

As illustrated in Table 3, the TLDCF-TSD-BBF model generally outperformed other variants across the provinces. For instance, in Heilongjiang, the full TLDCF-TSD-BBF model showed significant improvements over the BiTCEN-BiLSTM-FECAM model, with a 76.3% decrease in MAE, an 89.7% decrease in MSE, a 73.6% decrease in MAPE, and a 20.9% increase in R-squared. Removing the BiTCEN module led to a 3.9% increase in MAE, a 2.5% increase in MSE, a 34.8% increase in MAPE, and a 0.6% decrease in R-squared. Similarly, excluding the FECAM module resulted in a 3.5% increase in MAE, a 2.0% increase in MSE, a 0.7% increase in MAPE, and a 0.7% decrease in R-squared. These results highlight the importance of the TLDCF-TSD, BiTCEN, and FECAM modules in enhancing the model’s accuracy and stability. The radar chart (Fig 10) comparing the ablation models with the proposed model visually demonstrates that the omission of any component leads to a decline in predictive performance, thereby validating the contribution of each module to the model’s precision and robustness.

**Table 3:** Specific predictive performance indicators of the comparison model and the model proposed in this paper.

Province	Models	MAE	MSE	MAPE	R-squared
Heilongjiang	TLDCF-TSD-BBF	0.0093	0.0002	1.3630	0.9891
	BiTCEN-BiLSTM-FECAM	0.0405	0.0030	5.3721	0.8107
	TLDCF-TSD-BiLSTM-FECAM	0.0107	0.0006	1.4212	0.9809
	TLDCF-TSD-BiTCEN-BiLSTM	0.0153	0.0006	1.9159	0.9679
Jilin	TLDCF-TSD-BBF	0.0137	0.0002	1.7456	0.9888
	BiTCEN-BiLSTM-FECAM	0.0413	0.0028	6.1453	0.8603
	TLDCF-TSD-BiLSTM-FECAM	0.0141	0.0002	1.9791	0.9886
	TLDCF-TSD-BiTCEN-BiLSTM	0.0140	0.0003	1.9578	0.9879
Shandong	TLDCF-TSD-BBF	0.0081	0.0001	1.1905	0.9943
	BiTCEN-BiLSTM-FECAM	0.0285	0.0016	4.0007	0.9137
	TLDCF-TSD-BiLSTM-FECAM	0.0093	0.0002	1.3791	0.9896
	TLDCF-TSD-BiTCEN-BiLSTM	0.0090	0.0001	1.2427	0.9921
Henan	TLDCF-TSD-BBF	0.0055	0.0001	0.8456	0.9955
	BiTCEN-BiLSTM-FECAM	0.0198	0.0008	3.0007	0.9203
	TLDCF-TSD-BiLSTM-FECAM	0.0067	0.0006	1.0234	0.9934
	TLDCF-TSD-BiTCEN-BiLSTM	0.0059	0.0003	0.8879	0.9943
Shanxi	TLDCF-TSD-BBF	0.0101	0.0002	1.7567	0.9933
	BiTCEN-BiLSTM-FECAM	0.0403	0.0035	6.4481	0.8869
	TLDCF-TSD-BiLSTM-FECAM	0.0155	0.0008	2.2075	0.9874
	TLDCF-TSD-BiTCEN-BiLSTM	0.0102	0.0002	1.9441	0.9929

### Comparison experiment analysis

In time series data analysis, there are significant differences in the effectiveness of different models and data processing methods. In order to explore in depth the performance of different models in different regions and the advantages of specific module combinations, this comparative experiment was conducted. The experiment covers data from several provinces and compares the prediction performance of several deep learning models, with evaluation metrics including MAE, MSE, MAPE, and R-squared. Tables 4 and 5 show the experimental result data of each model in different provinces, from which we can clearly see the performance difference of each model on different regional data.

**Table 4:** Prediction accuracy indicators of the ablation model and the model proposed in this paper.

Province	Models	MAE	MSE	MAPE	R-squared
Heilongjiang	LSTM	0.0665	0.0085	8.5305	0.5477
	BiLSTM	0.0436	0.0039	5.5151	0.7917
	TLDCF-TSD-BiLSTM	0.0180	0.0009	1.9212	0.9623
	CNN-BiLSTM	0.0541	0.0065	6.2490	0.7057
	CNN-BiLSTM-Attention	0.0505	0.0054	6.1297	0.7118
	TCN-BiLSTM	0.0591	0.0077	7.0883	0.5928
	TCN-BiLSTM-Attention	0.0589	0.0078	7.0296	0.5860
	TLDCF-TSD-TCN-BiLSTM	0.0206	0.0012	2.4099	0.9317
	BiTCEN-BiLSTM	0.0425	0.0037	5.4391	0.7975
	BiTCEN-BiLSTM-Attention	0.0535	0.0061	6.5134	0.6755
	TLDCF-TSD-BiTCEN-BiLSTM-Attention	0.0143	0.0005	1.7154	0.9712
	TLDCF-TSD-BBF	0.0093	0.0002	1.3630	0.9891
Jilin	LSTM	0.0694	0.0080	10.6636	0.7395
	BiLSTM	0.0444	0.0040	6.6404	0.8695
	TLDCF-TSD-BiLSTM	0.0246	0.0013	4.0923	0.9564
	CNN-BiLSTM	0.0510	0.0048	7.2570	0.8441
	CNN-BiLSTM-Attention	0.0511	0.0048	7.2808	0.8434
	TCN-BiLSTM	0.0535	0.0051	7.4871	0.8328
	TCN-BiLSTM-Attention	0.0526	0.0051	7.3554	0.8357
	TLDCF-TSD-TCN-BiLSTM	0.0230	0.0009	3.0143	0.9656
	BiTCEN-BiLSTM	0.0442	0.0035	6.6361	0.8729
	BiTCEN-BiLSTM-Attention	0.0552	0.0055	7.6965	0.8226
	TLDCF-TSD-BiTCEN-BiLSTM-Attention	0.0158	0.0003	1.7744	0.9857
	TLDCF-TSD-BBF	0.0137	0.0002	1.7456	0.9888
Shandong	LSTM	0.0421	0.0045	6.0991	0.7561
	BiLSTM	0.0328	0.0021	4.3903	0.8931
	TLDCF-TSD-BiLSTM	0.0192	0.0007	3.2995	0.9617
	CNN-BiLSTM	0.0312	0.0018	4.0637	0.9032
	CNN-BiLSTM-Attention	0.0337	0.0020	4.6304	0.8895
	TCN-BiLSTM	0.0317	0.0021	4.4197	0.9020
	TCN-BiLSTM-Attention	0.0305	0.0019	4.0939	0.9046
	TLDCF-TSD-TCN-BiLSTM	0.0164	0.0004	2.2531	0.9756
	BiTCEN-BiLSTM	0.0292	0.0018	4.0096	0.9132
	BiTCEN-BiLSTM-Attention	0.0294	0.0017	4.0993	0.9037
	TLDCF-TSD-BiTCEN-BiLSTM-Attention	0.0084	0.0001	1.2716	0.9925
	TLDCF-TSD-BBF	0.0081	0.0001	1.1905	0.9943

**Table 5:** Prediction accuracy indicators of the ablation model and the model proposed in this paper.

Province	Models	MAE	MSE	MAPE	R-squared
Henan	LSTM	0.0404	0.0048	6.2600	0.5707
	BiLSTM	0.0219	0.0010	3.4491	0.9098
	TLDCF-TSD-BiLSTM	0.0074	0.0005	1.8414	0.9745
	CNN-BiLSTM	0.0213	0.0010	3.2168	0.9106
	CNN-BiLSTM-Attention	0.0207	0.0010	3.1495	0.9108
	TCN-BiLSTM	0.0216	0.0010	3.3580	0.9093
	TCN-BiLSTM-Attention	0.0209	0.0009	3.2210	0.9117
	TLDCF-TSD-TCN-BiLSTM	0.0098	0.0002	1.4781	0.9831
	BiTCEN-BiLSTM	0.0204	0.0009	3.1345	0.9115
	BiTCEN-BiLSTM-Attention	0.0209	0.0009	3.1867	0.9114
	TLDCF-TSD-BiTCEN-BiLSTM-Attention	0.0065	0.0002	0.9597	0.9864
	TLDCF-TSD-BBF	0.0055	0.0001	0.8456	0.9955
Shanxi	LSTM	0.0526	0.0059	9.3859	0.8021
	BiLSTM	0.0461	0.0044	8.2208	0.8522
	TLDCF-TSD-BiLSTM	0.0174	0.0007	2.7153	0.9825
	CNN-BiLSTM	0.0422	0.0041	6.7651	0.8600
	CNN-BiLSTM-Attention	0.0415	0.0039	6.9806	0.8676
	TCN-BiLSTM	0.0436	0.0040	7.2765	0.8640
	TCN-BiLSTM-Attention	0.0425	0.0039	7.1241	0.8684
	TLDCF-TSD-TCN-BiLSTM	0.0143	0.0004	2.2863	0.9841
	BiTCEN-BiLSTM	0.0424	0.0039	7.0934	0.8692
	BiTCEN-BiLSTM-Attention	0.0418	0.0038	6.9937	0.8730
	TLDCF-TSD-BiTCEN-BiLSTM-Attention	0.0103	0.0002	1.8507	0.9904
	TLDCF-TSD-BBF	0.0101	0.0002	1.7567	0.9933

In the experimental results of Heilongjiang Province, the traditional LSTM model demonstrated an MAE of 0.0665, an MSE of 0.0085, and a MAPE of 8.5305, and an R-squared of 0.5477. In contrast, the BiLSTM model demonstrates marginal enhancement, with an MAE of 0.0436, an MSE of 0.0039, a MAPE of 5.5151, and an R-squared of 0.7917. The BiLSTM model has been calculated to reduce the MAE by approximately 34.44%, the MSE by approximately 54.12%, the MAPE by approximately 35.35%, and the R-squared by approximately 44.44%, relative to the traditional LSTM model. The squared term has been improved by approximately 44.55%.Incorporating the TLDCF-TSD module, as demonstrated by the TLDCF-TSD-BiLSTM model, results in a substantial reduction in MAE, MSE, and MAPE by approximately 58.72%, in comparison with the BiLSTM model. Moreover, the MSE is reduced by around 76.92%, the MAPE is decreased by approximately 65.17%, and concurrently, the R-squared is enhanced by about 21.55%. This unequivocally substantiates that the module significantly enhances the model’s performance.In the context of models integrated with alternative modules, CNN - BiLSTM and CNN - BiLSTM - Attention exhibited relatively elevated error metrics, with values of 0.7057 and 0.7118, respectively. In contrast, TCN - BiLSTM and TCN - BiLSTM - Attention demonstrated performance that was not significantly superior to that of certain alternative models, with MAEs of 0.0591 and 0.0589, respectively. The BiTCEN - BiLSTM and BiTCEN - BiLSTM - Attention models demonstrate some degree of performance, though not to the same level as certain models equipped with TLDCF - TSD modules. Notably, the TLDCF-TSD-BiTCEN-BiLSTM-Attention model demonstrates particularly strong performance, with an MAE of 0.0143, an MSE of 0.0005, a MAPE of 1.7154, and an R of 0.9712. A comparison of this model with some of the previously mentioned models reveals significant improvements in various indicators. Specifically, the MAE is reduced by approximately 75.77% in comparison with the CNN-BiLSTM model.In addition, the TLDCF - TSD - BBF model demonstrates notable efficacy in the context of Heilongjiang province, exhibiting an MAE of 0.0093, an MSE of 0.0002, a MAPE of 1.3630, and an R-squared of 0.9891. This represents a 34.97% reduction in MAE and a 34.97% reduction in MSE when compared with the TLDCF - TSD - BiTCEN - BiLSTM - Attention model. In comparison with the TLDCF-TSD-BiTCEN-BiLSTM-Attention model, the percentage reduction of MAE is approximately 34.97%, the percentage reduction of MSE is approximately 60%, the percentage reduction of MAPE is approximately 20.54%, and the percentage increase of R-squared is approximately 1.84%.

**Fig 11:**
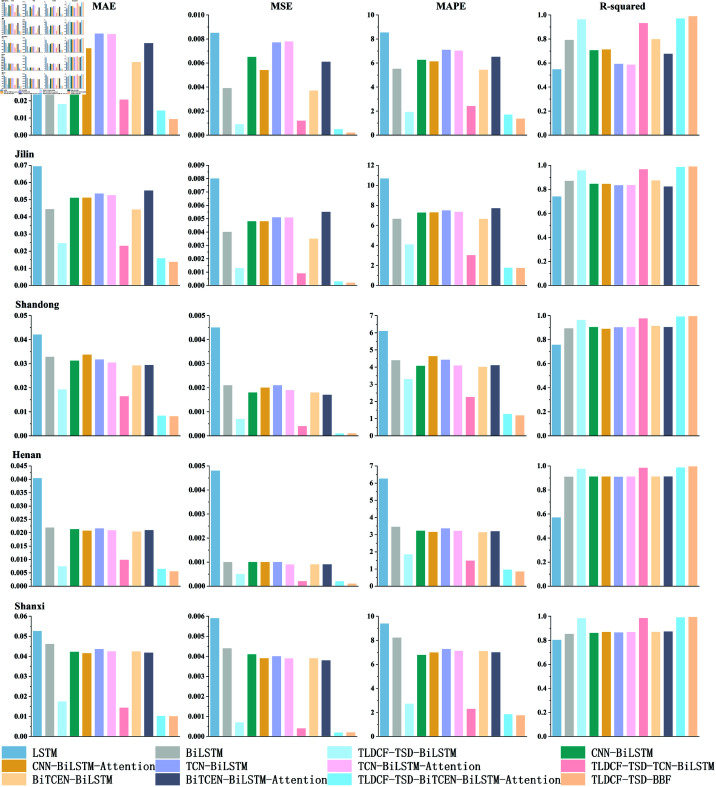
Bar chart comparing the prediction accuracy indicators of the model and the model proposed in this paper.

**Fig 12:**
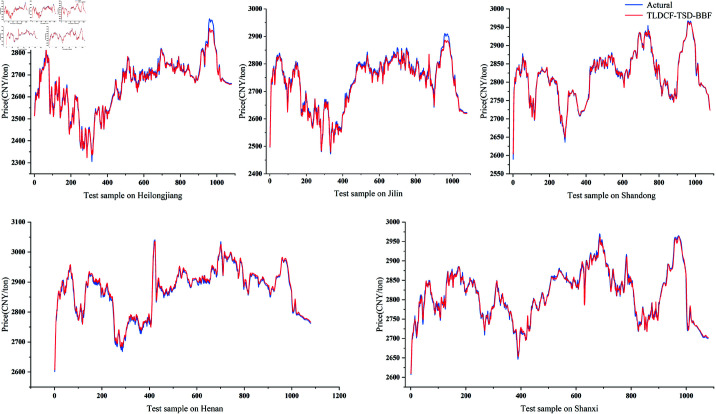
Prediction results of our model compared to the actual values.

The other four provinces show similar trends. For example, in Jilin province, the TLDCF-TSD-BBF model also performs well on all indicators, with lower error and higher goodness of fit than the other models. Fig 11 shows the results of the visualisation of the experimental data in each province, which makes it possible to intuitively compare the differences in the predictive effects of different models, as well as the comparative performance of the models on the various evaluation indicators. The prediction results of TLDCF-TSD-BBF are shown in Fig 12. Although there were significant differences in the experimental results of Heilongjiang and Jilin under certain special circumstances, overall, the basic trends of the two are the same.

Taken together, the TLDCF-TSD-BBF model shows obvious advantages in most of the evaluation indices in predicting corn price time series data in five provinces, which demonstrates its excellent predictive ability in dealing with this kind of data, and also shows the positive effect of TLDCF-TSD and other related modules in improving the performance of the model.

### DM test

In order to verify the prediction performance of the models, the DM test was conducted in this paper, and the results are shown in the following table. In the DM test, the DM statistics of each model were calculated based on the three prediction error assessment indexes of MAE, MSE and MAPE, using provincial data under different conditions. As shown in Tables 6 and 7, the DM statistics of the LSTM and BiLSTM models are significantly negative in the data of the Heilongjiang province, indicating that these models have greater errors in predicting corn prices compared with the TLDCF-TSD-BBF model. A similar pattern is observed in the data from Jilin, Shandong, Henan and Shanxi provinces, where the DM statistics of different models are different, but the overall trend is the same. This suggests that when comparing the TLDCF-TSD-BBF model proposed in this paper with its related variants and other comparative models, the difference in the performance of the TLDCF-TSD-BBF model in predicting maize price is reflected by the DM test statistic. This further proves that the TLDCF-TSD-BBF model can predict maize price more accurately than other models. The TLDCF-TSD-BBF model has been demonstrated to exhibit superior predictive capabilities and increased reliability in comparison to the other 14 models.

**Table 6:** DM test results of different models for forecasting.

Province	Model	DM1	DM2	DM3
Heilongjiang	LSTM	-22.4748^***^	-13.7040^***^	-18.3018^***^
	BiLSTM	-23.2441^***^	-15.3978^***^	-18.9849^***^
	TLDCF-TSD-BiLSTM	-4.9068^***^	-7.5122^***^	-6.0545^***^
	CNN-BiLSTM	-23.5603^***^	-13.5094^***^	-19.3164^***^
	CNN-BiLSTM-Attention	-25.4117^***^	-15.8563^***^	-22.2110^***^
	TCN-BiLSTM	-22.5623^***^	-14.1266^***^	-18.1515^***^
	TCN-BiLSTM-Attention	-22.1861^***^	-14.3734^***^	-19.1996^***^
	TLDCF-TSD-TCN-BiLSTM	-16.8968^***^	-11.8851^***^	-16.1579^***^
	BiTCEN-BiLSTM	-23.3083^***^	-15.2729^***^	-19.1567^***^
	BiTCEN-BiLSTM-Attention	-24.4948^***^	-15.6648^***^	-20.7191^***^
	TLDCF-TSD-BiTCEN-BiLSTM-Attention	-2.4430^**^	-5.2611^***^	-0.6472
	BiTCEN-BiLSTM-FECAM	-27.3524^***^	-18.9500^***^	-20.7516^***^
	TLDCF-TSD-BiLSTM-FECAM	-3.2187^***^	-5.6277^***^	-5.0699^***^
	TLDCF-TSD-BiTCEN-BiLSTM	-1.0990	-4.1039^***^	-0.5294
Jilin	LSTM	-26.0981^***^	-18.0341^***^	-22.5620^***^
	BiLSTM	-20.5899^***^	-13.1885^***^	-17.1108^***^
	TLDCF-TSD-BiLSTM	-7.9935^***^	-6.9899^***^	-7.8601^***^
	CNN-BiLSTM	-26.2981^***^	-18.1808^***^	-21.3284^***^
	CNN-BiLSTM-Attention	-25.1033^***^	-18.3871^***^	-19.9525^***^
	TCN-BiLSTM	-22.7203^***^	-13.5560^***^	-15.8724^***^
	TCN-BiLSTM-Attention	-24.4577^***^	-17.3772^***^	-17.4904^***^
	TLDCF-TSD-TCN-BiLSTM	-20.3235^***^	-15.5850^***^	-14.2850^***^
	BiTCEN-BiLSTM	-24.1176^***^	-18.1569^***^	-19.2821^***^
	BiTCEN-BiLSTM-Attention	-23.8405^***^	-17.4970^***^	-19.6277^***^
	TLDCF-TSD-BiTCEN-BiLSTM-Attention	-4.9150^***^	-2.7773^***^	-4.3791^***^
	BiTCEN-BiLSTM-FECAM	-23.4746^***^	-16.3588^***^	-18.3921^***^
	TLDCF-TSD-BiLSTM-FECAM	-4.2614^***^	-3.2483^***^	-5.9650^***^
	TLDCF-TSD-BiTCEN-BiLSTM	-4.0793^***^	-3.5466^***^	-4.2015^***^
Shandong	LSTM	-27.4871^***^	-16.9161^***^	-26.6073^***^
	BiLSTM	-21.7900^***^	-11.2242^***^	-20.0759^***^
	TLDCF-TSD-BiLSTM	-16.7769^***^	-9.5838^***^	-15.1497^***^
	CNN-BiLSTM	-28.9800^***^	-20.5743^***^	-29.5658^***^
	CNN-BiLSTM-Attention	-26.9315^***^	-18.9965^***^	-26.2141^***^
	TCN-BiLSTM	-24.0969^***^	-11.0191^***^	-20.0925^***^
	TCN-BiLSTM-Attention	-23.9030^***^	-12.6891^***^	-19.8648^***^
	TLDCF-TSD-TCN-BiLSTM	-20.578^***^	-8.9184^***^	-17.1927^***^
	BiTCEN-BiLSTM	-26.9381^***^	-18.7831^***^	-25.8841^***^
	BiTCEN-BiLSTM-Attention	-25.8347^***^	-18.4138^***^	-24.9704^***^
	TLDCF-TSD-BiTCEN-BiLSTM-Attention	-6.4293^***^	-5.6179^***^	-5.6599^***^
	BiTCEN-BiLSTM-FECAM	-26.3023^***^	-17.8458^***^	-24.8425^***^
	TLDCF-TSD-BiLSTM-FECAM	-9.8439^***^	-7.4565^***^	-8.9643^***^
	TLDCF-TSD-BiTCEN-BiLSTM	-15.7042^***^	-14.4475^***^	-15.0251^***^

**Note:** ^*^, ^**^, and ^***^ denote rejection of the null hypothesis at the 10%, 5%, and 1% significance levels respectively. DM1, DM2, and DM3 denote the values of the DM test statistic under the MAE, MSE, and MAPE prediction error evaluation metrics respectively.

**Table 7:** DM test results of different models for forecasting.

Province	Model	DM1	DM2	DM3
Henan	LSTM	-26.5412^***^	-14.9100^***^	-23.9496^***^
	BiLSTM	-16.5933^***^	-11.7886^***^	-15.6953^***^
	TLDCF-TSD-BiLSTM	-3.1869^***^	-5.1549^***^	-2.4709^***^
	CNN-BiLSTM	-18.7778^***^	-10.2513^***^	-18.5699^***^
	CNN-BiLSTM-Attention	-17.9334^***^	-12.8032^***^	-16.6561^***^
	TCN-BiLSTM	-16.9973^***^	-10.0383^***^	-13.6217^***^
	TCN-BiLSTM-Attention	-16.1643^***^	-10.3653^***^	-13.3267^***^
	TLDCF-TSD-TCN-BiLSTM	-14.2100^***^	-8.4161^***^	-10.7441^***^
	BiTCEN-BiLSTM	-19.3486^***^	-12.6770^***^	-17.8859^***^
	BiTCEN-BiLSTM-Attention	-16.5877^***^	-11.5963^***^	-14.3503^***^
	TLDCF-TSD-BiTCEN-BiLSTM-Attention	-2.1334^**^	-0.1195	-2.4888^**^
	BiTCEN-BiLSTM-FECAM	-16.6278^***^	-11.5169^***^	-15.3319^***^
	TLDCF-TSD-BiLSTM-FECAM	-2.5538^**^	-0.9087	-3.2614^***^
	TLDCF-TSD-BiTCEN-BiLSTM	-5.4068^***^	-4.5107^***^	-6.5016^***^
Shanxi	LSTM	-27.3407^***^	-13.6733^***^	-23.1617^***^
	BiLSTM	-27.9497^***^	-14.8722^***^	-24.9527^***^
	TLDCF-TSD-BiLSTM	-10.6281^***^	-9.0281^***^	-9.5007^***^
	CNN-BiLSTM	-26.1033^***^	-14.1178^***^	-23.7912^***^
	CNN-BiLSTM-Attention	-20.5028^***^	-9.0319^***^	-18.2516^***^
	TCN-BiLSTM	-23.7312^***^	-13.4116^***^	-19.0038^***^
	TCN-BiLSTM-Attention	-23.1142^***^	-13.2153^***^	-18.6332^***^
	TLDCF-TSD-TCN-BiLSTM	-10.8536^***^	-6.6094^***^	-7.7758^***^
	BiTCEN-BiLSTM	-22.2941^***^	-12.8049^***^	-20.6081^***^
	BiTCEN-BiLSTM-Attention	-22.2430^***^	-11.4686^***^	-20.3064^***^
	TLDCF-TSD-BiTCEN-BiLSTM-Attention	-3.5459^***^	-2.1267^**^	-3.0312^***^
	BiTCEN-BiLSTM-FECAM	-22.3712^***^	-12.4971^***^	-21.0142^***^
	TLDCF-TSD-BiLSTM-FECAM	-3.4403^***^	-3.7762^***^	-3.6115^***^
	TLDCF-TSD-BiTCEN-BiLSTM	-9.5198^***^	-7.5601^***^	-8.9145^***^

**Note:** ^*^, ^**^, and ^***^ denote rejection of the null hypothesis at the 10%, 5%, and 1% significance levels respectively. DM1, DM2, and DM3 denote the values of the DM test statistic under the MAE, MSE, and MAPE prediction error evaluation metrics respectively.

### Robustness check

A reliable model should demonstrate the capacity to function consistently under varying conditions of the test set. In this study of corn price prediction, an initial 8:2 ratio of training and test sets was utilised for model construction and evaluation. However, in order to explore the model’s performance more comprehensively under different data volume scenarios and to assess its generalisation ability, we adjusted the training to test set ratio to 5:5.

The ensuing Tables 8 and 8 presents the DM test results of each model utilised to predict corn prices following the adjustment of the training set to test set ratio to 5:5. It is evident that the DM statistics of all models are negative, and the majority of them reject the null hypothesis at the 1% significance level. This outcome indicates that the TLDCF - TSD - BBF model proposed in this study exhibits robust performance under varying proportions of data distribution, i.e., the model demonstrates considerable resilience in the face of different data volumes. This suggests that the model can effectively extract meaningful information from the corn price data, regardless of changes in data volume. Consequently, it can provide more accurate price forecasts, thereby supporting informed decision-making in the corn market.

**Table 8:** DM test results of different models for forecasting(training set: test set = 5:5).

Province	Model	DM1	DM2	DM3
Heilongjiang	LSTM	-24.4999^***^	-14.4619^***^	-26.5437^***^
	BiLSTM	-23.0549^***^	-18.3302^***^	-18.6753^***^
	TLDCF-TSD-BiLSTM	-2.3912^**^	-1.9752^**^	-2.2141^**^
	CNN-BiLSTM	-16.9643^***^	-8.5885^***^	-17.5184^***^
	CNN-BiLSTM-Attention	-26.8895^***^	-13.9081^***^	-21.8431^***^
	TCN-BiLSTM	-23.5362^***^	-13.3357^***^	-19.5950^***^
	TCN-BiLSTM-Attention	-23.4671^***^	-13.3171^***^	-20.4822^***^
	TLDCF-TSD-TCN-BiLSTM	-23.5996^***^	-12.9712^***^	-19.4079^***^
	BiTCEN-BiLSTM	-26.1721^***^	-15.0462^***^	-23.5412^***^
	BiTCEN-BiLSTM-Attention	-25.6562^***^	-17.2604^***^	-20.9445^***^
	TLDCF-TSD-BiTCEN-BiLSTM-Attention	-1.6677^*^	-2.9043^***^	-1.8436^*^
	BiTCEN-BiLSTM-FECAM	-23.7034^***^	-18.2504^***^	-18.4720^***^
	TLDCF-TSD-BiLSTM-FECAM	-5.8998^***^	-1.9477^*^	-6.6294^***^
	TLDCF-TSD-BiTCEN-BiLSTM	-9.2003^***^	-6.6936^***^	-6.8766^***^
Jilin	LSTM	-27.3150^***^	-16.9073^***^	-25.3421^***^
	BiLSTM	-22.4637^***^	-17.4538^***^	-19.3546^***^
	TLDCF-TSD-BiLSTM	-4.6906^***^	-4.5091^***^	-5.8872^***^
	CNN-BiLSTM	-22.8162^***^	-16.4462^***^	-18.4298^***^
	CNN-BiLSTM-Attention	-24.2943^***^	-17.6791^***^	-19.1006^***^
	TCN-BiLSTM	-22.3702^***^	-15.4241^***^	-17.7189^***^
	TCN-BiLSTM-Attention	-22.5578^***^	-16.1003^***^	-18.4312^***^
	TLDCF-TSD-TCN-BiLSTM	-15.6889^***^	-11.7944^***^	-13.2580^***^
	BiTCEN-BiLSTM	-24.5399^***^	-17.2284^***^	-21.5178^***^
	BiTCEN-BiLSTM-Attention	-23.9493^***^	-17.8256^***^	-20.1516^***^
	TLDCF-TSD-BiTCEN-BiLSTM-Attention	-2.3426^**^	-1.7759^*^	-2.0572^**^
	BiTCEN-BiLSTM-FECAM	-23.0151^***^	-16.3464^***^	-18.7878^***^
	TLDCF-TSD-BiLSTM-FECAM	-6.3118^***^	-5.4286^***^	-7.4924^***^
	TLDCF-TSD-BiTCEN-BiLSTM	-2.3698^**^	-1.6016	-1.9664^**^
Shandong	LSTM	-29.0721^***^	-17.0754^***^	-27.8535^***^
	BiLSTM	-11.2472^***^	-6.3165^***^	-13.0593^***^
	TLDCF-TSD-BiLSTM	-1.9618^**^	-3.7783^***^	-1.8623^*^
	CNN-BiLSTM	-25.7196^***^	-15.2430^***^	-26.5880^***^
	CNN-BiLSTM-Attention	-26.0831^***^	-15.3318^***^	-27.2994^***^
	TCN-BiLSTM	-22.9305^***^	-14.1084^***^	-22.9855^***^
	TCN-BiLSTM-Attention	-24.1183^***^	-14.3573^***^	-24.1505^***^
	TLDCF-TSD-TCN-BiLSTM	-20.1385^***^	-12.8829^***^	-19.1115^***^
	BiTCEN-BiLSTM	-22.9369^***^	-13.6075^***^	-25.1490^***^
	BiTCEN-BiLSTM-Attention	-23.3145^***^	-13.9160^***^	-25.7981^***^
	TLDCF-TSD-BiTCEN-BiLSTM-Attention	-14.9034^***^	-11.7094^***^	-14.8860^***^
	BiTCEN-BiLSTM-FECAM	-25.1262^***^	-15.2142^***^	-26.5210^***^
	TLDCF-TSD-BiLSTM-FECAM	-2.0227^**^	-2.6146^***^	-3.0963^***^
	TLDCF-TSD-BiTCEN-BiLSTM	-1.7417^*^	-5.1761^***^	-1.8422^*^

**Note:** ^*^, ^**^, and ^***^ denote rejection of the null hypothesis at the 10%, 5%, and 1% significance levels respectively. DM1, DM2, and DM3 denote the values of the DM test statistic under the MAE, MSE, and MAPE prediction error evaluation metrics respectively.

**Table 9:** DM test results of different models for forecasting(training set: test set = 5:5).

Province	Model	DM1	DM2	DM3
Henan	LSTM	-23.6298^***^	-14.4926^***^	-24.2754^***^
	BiLSTM	-19.4338^***^	-13.4205^***^	-19.0699^***^
	TLDCF-TSD-BiLSTM	-1.9787^**^	-4.7113^***^	-3.2985^***^
	CNN-BiLSTM	-19.4359^***^	-12.0243^***^	-18.8829^***^
	CNN-BiLSTM-Attention	-21.5174^***^	-14.7342^***^	-19.3447^***^
	TCN-BiLSTM	-22.4321^***^	-14.3363^***^	-18.9153^***^
	TCN-BiLSTM-Attention	-19.8782^***^	-12.4975^***^	-16.8884^***^
	TLDCF-TSD-TCN-BiLSTM	-15.0403^***^	-9.3724^***^	-11.5003^***^
	BiTCEN-BiLSTM	-23.8339^***^	-16.3473^***^	-22.3926^***^
	BiTCEN-BiLSTM-Attention	-21.3075^***^	-14.3004^***^	-19.4103^***^
	TLDCF-TSD-BiTCEN-BiLSTM-Attention	-9.6305^***^	-10.0952^***^	-8.0953^***^
	BiTCEN-BiLSTM-FECAM	-20.5942^***^	-13.7095^***^	-19.2463^***^
	TLDCF-TSD-BiLSTM-FECAM	-1.6871^*^	-2.5447^**^	-3. 5519^***^
	TLDCF-TSD-BiTCEN-BiLSTM	-5.3375^***^	-5.5681^***^	-4.7371^***^
Shanxi	LSTM	-23.8122^***^	-13.4952^***^	-21.2536^***^
	BiLSTM	-17.8833^***^	-10.0325^***^	-17.9205^***^
	TLDCF-TSD-BiLSTM	-4.3053^***^	-1.6858^*^	-4.1278^***^
	CNN-BiLSTM	-17.7385^***^	-7.5688^***^	-15.1085^***^
	CNN-BiLSTM-Attention	-19.8226^***^	-9.8887^***^	-17.3708^***^
	TCN-BiLSTM	-23.7141^***^	-14.1224^***^	-21.9401^***^
	TCN-BiLSTM-Attention	-27.8567^***^	-17.0901^***^	-24.0838^***^
	TLDCF-TSD-TCN-BiLSTM	-16.1063^***^	-11.2103^***^	-13.7391^***^
	BiTCEN-BiLSTM	-22.7434^***^	-14.3112^***^	-20.6081^***^
	BiTCEN-BiLSTM-Attention	-21.7192^***^	-13.1772^***^	-22.2462^***^
	TLDCF-TSD-BiTCEN-BiLSTM-Attention	-4.8133^***^	-5.4652^***^	-3.3927^***^
	BiTCEN-BiLSTM-FECAM	-20.0569^***^	-9.3172^***^	-18.6334^***^
	TLDCF-TSD-BiLSTM-FECAM	-4.2073^***^	-5.3824^***^	-2.3103^**^
	TLDCF-TSD-BiTCEN-BiLSTM	-3.4406^***^	-5.1982^***^	-1.9907^**^

**Note:** ^*^, ^**^, and ^***^ denote rejection of the null hypothesis at the 10%, 5%, and 1% significance levels respectively. DM1, DM2, and DM3 denote the values of the DM test statistic under the MAE, MSE, and MAPE prediction error evaluation metrics respectively.

## Conclusion

This study introduces a novel hybrid model, TLDCF-TSD-BBF, which integrates advanced signal processing and deep learning techniques for the accurate prediction of corn prices, thereby enhancing the sustainability of the corn industry. The model demonstrates superior performance across multiple provinces, outperforming existing baseline models on various evaluation metrics. The proposed model combines TLDCF-TSD, BiTCEN, BiLSTM, and FECAM to predict corn prices. It has shown exceptional performance in forecasting tasks across various provinces, outperforming baseline models such as LSTM, BiLSTM, CNN-BiLSTM, TCN-BiLSTM, and their variants. Furthermore, an experiment was conducted in which the ratio of the training set to the testing set in corn price prediction was altered. This experiment confirmed that the model proposed in this paper exhibited excellent robustness, that is to say, it proved to be very reliable when the dataset and the amount of data were changed. The detailed summary of the research results is as follows:

1. TLDCF-TSD avoids noise masking or interfering with data trends and features, reduces data irregularity and clutter, and provides rich and high-quality data for the corn price prediction model to improve the model’s predictive ability.
2. TLDCF-TSD-BBF extracts the depth information more completely than other forecasting models, which is superior and has better forecasting results compared to other models.
3. BiTCEN is able to extract rich and accurate features more effectively through its unique bidirectional structure design, combined with forward and backward time convolution layer processing and optimised activation function and convolution combinations, which improves the model’s ability to process complex time series data and its prediction performance.

In summary, while the TLDCF-TSD-BBF model is designed for corn price prediction, its universality, due to its multi-scale feature extraction and deep information processing capabilities, makes it extensible to other agricultural product price predictions and various time series forecasts. Future work includes expanding the model to incorporate internal and external exogenous factors affecting prices, such as weather patterns, market dynamics, and policy changes, to further enhance its generalization capability and predictive accuracy. This advancement is of significant reference value and practical significance for farmers, policymakers, and market participants in making informed decisions, thereby promoting the sustainability of the corn industry.

Furthermore, the study demonstrates the immense potential of combining advanced signal processing techniques with deep learning models in the field of agricultural product price forecasting. It provides a new perspective and tools for research and practice in related fields, contributing to the sustainable development of the agricultural sector.

## Supporting information

S1 Text Experimental sample data (RAR)
